# Localization of cassava brown streak virus in *Nicotiana rustica* and cassava *Manihot esculenta* (Crantz) using RNAscope® in situ hybridization

**DOI:** 10.1186/s12985-018-1038-z

**Published:** 2018-08-14

**Authors:** Esperance Munganyinka, Paolo Margaria, Samar Sheat, Elijah M. Ateka, Fred Tairo, Joseph Ndunguru, Stephan Winter

**Affiliations:** 1 Rwanda Agriculture Board, P.O. Box 5016, Kigali, Rwanda; 2 Leibniz Institute DSMZ-German Collection of Microorganisms and Cell Cultures, Plant Virus Department, Messeweg 11/12, 38104 Braunschweig, Germany; 3 Jomo Kenyatta University of Agriculture and Technology, P.O. Box 62000-00200, Nairobi, Kenya; 4 Mikocheni Agricultural Research Institute, P.O. Box 6226, Dar es Salaam, Tanzania

**Keywords:** Cassava brown streak virus, Virus localization, Ipomovirus, In situ hybridization, RNAscope®, Formalin-fixed paraffin-embedded tissue sections

## Abstract

**Background:**

Cassava brown streak disease (CBSD) has a viral aetiology and is caused by viruses belonging to the genus *Ipomovirus* (family *Potyviridae*), *Cassava brown streak virus* (CBSV) and *Ugandan cassava brown streak virus* (UCBSV). Molecular and serological methods are available for detection, discrimination and quantification of cassava brown streak viruses (CBSVs) in infected plants. However, precise determination of the viral RNA localization in infected host tissues is still not possible pending appropriate methods.

**Results:**

We have developed an in situ hybridization (ISH) assay based on RNAscope® technology that allows the sensitive detection and localization of CBSV RNA in plant tissues. The method was initially developed in the experimental host *Nicotiana rustica* and was then further adapted to cassava. Highly sensitive and specific detection of CBSV RNA was achieved without background and hybridization signals in sections prepared from non-infected tissues. The tissue tropism of CBSV RNAs appeared different between *N. rustica* and cassava.

**Conclusions:**

This study provides a robust method for CBSV detection in the experimental host and in cassava. The protocol will be used to study CBSV tropism in various cassava genotypes, as well as CBSVs/cassava interactions in single and mixed infections.

## Background

Cassava brown streak disease (CBSD) is caused by two distinct virus species, *Cassava brown streak virus* (CBSV) and *Ugandan cassava brown streak virus* (UCBSV), both members of the genus *Ipomovirus* in the family *Potyviridae* [1]. The viruses are the most devastating pathogens of cassava (*Manihot esculenta*) in Africa, threatening cassava cultivation particularly in East and Central Africa [2]. The viruses have single-stranded RNA genomes of about 9000 nt, and while genetically distinct, they cause similar symptoms in the leaves, stems and root tissues of cassava [1, 3, 4] including leaf chlorosis, brown streaks on stems and necrosis of root tubers [5]. The natural host range of CBSVs is restricted to *M. esculenta* and *Manihot glaziovii*, a perennial species related to cassava, but other natural hosts which could serve as viral inoculum sources may exist [2, 6].

Immunological and molecular techniques for the detection of CBSVs have been developed. Enzyme-linked immunosorbent assays (ELISA) based on monoclonal antibodies [7], reverse transcription-polymerase chain reaction (RT-PCR) [8–10] and quantitative RT-PCR [11–14] are routinely used in diagnosis of the viruses. While these methods are sensitive, reproducible and robust for virus detection in a given cassava sample, accurate quantification of CBSVs in cassava is hampered by the uneven distribution of the virus in the plant [15, 16], making comparative studies very difficult.

The disease caused by cassava brown streak viruses is the subject of intensive research in many institutes around the world, and research on the causative viruses is a key topic in the DSMZ Plant Virus Department. In particular, we are interested in following the movement of cassava brown streak viruses in cassava to study tissue invasion and the possible association of CBSV with specific plant tissues and organs. This approach aims to investigate a possible correlation between the virus loads in leaf, stem and tuberous root tissues and the extent of necrotic brown streak symptoms in root tissues. In situ hybridization of CBSV RNA in cassava tissue sections requires highly sensitive methods to detect even small amounts of RNA. To address this aim, we have developed an in situ hybridization (ISH) method which is based on RNAscope® technology, allowing detection and localization of RNA targets with high specificity and sensitivity [17, 18]. The RNAscope® technology developed by Advanced Cell Diagnostics (ACD; Hayward, CA, USA) is based on a unique probe design and signal amplification strategy that results in high specificity and sensitivity. RNAscope® has been mostly used in clinical studies with human and animal tissues [17, 19–22]. Recently, two studies have also used RNAscope® in plant tissues: for the sensitive localization of messenger RNAs (mRNAs) coding for C4 photosynthetic enzymes in maize leaves [23] and for the simultaneous visualization of two isolates of *Citrus tristeza virus* (CTV) in the petioles and root tissues of citrus [24].

In this manuscript, we present a protocol for preparation of tissue sections from CBSV-infected *Nicotiana rustica* and cassava plants. We describe the optimal conditions for the ISH assay and provide a robust method for CBSV detection in tissue sections of its experimental host and cassava. The method represents a significant technical advancement enabling studies of CBSV-infected cassava organs and tissues to further advance our understanding of the mechanisms of CBSV infection and disease development.

## Methods

### Plant material and virus inoculations

The experimental host *N. rustica* and the cassava cultivar Tropical *Manihot esculenta* 7 (TME7) were used for this study. The plants were grown in a glasshouse at 24 to 26 °C. Virus infections were established using the virus isolate CBSV-Mo83 (DSMZ PV-0949), which has been isolated from naturally infected cassava collected in Mozambique [1]. CBSV-Mo83 was transmitted to *N. rustica* by mechanical inoculation. *N. rustica* plants at the three-to-four leaf stage were inoculated with inoculum prepared by grinding CBSV-Mo83-infected *N. benthamiana* leaves in 0.05 M phosphate buffer (0.05 M Na_3_PO_4_, 1 mM EDTA, 5 mM DIECA, 5 mM thioglycolic acid, pH 7.0) in a ratio of 1:20 (*w*/*v*). Symptoms developed after 7 days, and virus infection was confirmed by RT-PCR [1]. In cassava, CBSV-Mo83 was routinely maintained in var. TMS 96/0304 and propagated by cuttings or, for new infections, by grafting. Virus infections in TME7 were established by grafting buds of CBSV-Mo83-infected cassava var. TMS 96/0304 onto cassava plants grown from tissue culture. Virus infections were confirmed by symptom development and RT-PCR approximately four weeks after inoculation.

### Design and synthesis of CBSV-Mo83 probes

Probes against CBSV-Mo83 were custom-designed by ACD and are available in the ACD catalogue as V-CBSV-Mo83-P1 (Ref. 509,481). The probes were designed based on the CBSV-Mo83 genomic sequence (GenBank accession number FN434436) and were complementary to nucleotides at positions 127 to 1191 within the P1 sequence.

### Fixation, embedding, and sectioning of *N. rustica*

For the ISH assays of *N. rustica*, stem sections were prepared from healthy and CBSV-infected plants at 14 dpi. Stem tissues (~ 5 mm in diameter and length) were cut using a sterile razor blade and placed into 10% neutral buffered formalin fixative solution (Sigma-Aldrich, St. Louis, MO, USA). Incubation was performed for 45 min at room temperature (RT) under vacuum conditions, followed by a fixative exchange and a 45 min incubation period, after which the fixative was exchanged and the samples were incubated for 16 h. The samples were subsequently washed two times in DEPC-treated phosphate-buffered saline (PBS, pH 7.4) for 15 min and dehydrated by incubation in increasing ethanol concentrations (30%, 50%, 70%, 95%, 100%) for 30 min at each concentration. After dehydration, the tissues were directly embedded into a low-melting agarose solution (5% w/v) (Serva Electrophoresis GmbH, Heidelberg, Germany). The agarose was melted in a microwave and cooled to approximately 40 °C, and tissue samples were placed into the gel in the desired orientation. Semi-thin (10 μm) cross-sections were cut using a Microm HM 650 V vibrating blade microtome (Thermo-Fisher Scientific, Pittsburgh, PA, USA) and applied to Superfrost Plus slides (Thermo-Fisher Scientific). Sections were allowed to dry on the slides overnight at RT and then baked in a hybridization oven for 1 h at 60 °C. Dried slides were stored in a covered box with silica gel, before proceeding with the ISH assays.

### Fixation, embedding, and sectioning of cassava

Leaf, stem and petiole explants (~ 5 mm in length; stems diameter: ~ 4 mm; petioles diameter: ~ 1.5 mm) from healthy and CBSV-infected cassava were fixed following the same procedures as described for *N. rustica*. For infected plants, leaf and petiole samples were collected from symptomatic leaves showing chlorosis, and stem samples from stems showing brown streaks. Because of the nature of cassava stem tissues (hard cortex and soft central medulla), different embedding media were tested, including low-melting agarose (SERVA Electrophoresis GmbH, Heidelberg, Germany), low-melting polyester-wax (Plano GmbH, Wetzlar, Germany) and Paraffin Paraplast Plus (Sigma-Aldrich). Embedding of cassava samples in low-melting agarose and tissue sectioning was performed as described above for *N. rustica*. For embedding in low-melting wax, the tissues were infiltrated with wax using increasing concentrations of wax solubilized in ethanol. Tissues were incubated in ethanol/wax mixtures (2:1, 1:1, 1:2 (v/v), pure wax) at 40 °C for 1 h at each concentration and then transferred into pure low-melting polyester wax in Peel-A-Way molds (Sigma-Aldrich). Embedding of tissue samples in Paraplast Plus paraffin was performed using a sequential-steps protocol by infiltrating plant tissues at RT in ethanol/xylene mixtures at 2:1, 1:1, and 1:2 (v/v) and pure xylene for 45 min in each substitute mixture. The tissues were then infiltrated with xylene/paraffin substitutes at 2:1, 1:1, 1:2 (v/v), followed by pure paraffin, with 1 h at each step in an oven at 60 °C. The samples were then transferred and embedded into pure paraffin in Peel-A-Way molds. Samples in low-melting wax and paraffin molds were allowed to cool to RT and stored at 4 °C overnight prior to sectioning. The blocks were trimmed to a suitable size, and cross-sections of 10–15 μm were prepared using a Microm HM 355 rotary microtome (Thermo-Fisher Scientific). After sectioning, the obtained ribbons were placed in a water bath at 37 °C and then placed on Superfrost Plus slides. Sections were completely dried and baked for 1 h at 60 °C. After baking, sections were deparaffinized in xylene (two times, 5 min each wash), washed in absolute ethanol, and stored in a covered box with silica gel before proceeding with the ISH assays.

### Optimization of RNAscope® ISH procedure

The ISH assay was performed using the ACD RNAscope® 2.5 HD Detection Reagent-RED kit (cat. no. 322360). A reference RNAscope® hybridization protocol provided by ACD (http://www.acdbio.com/technical-support/user-manuals) was essentially followed by modifying the pre-hybridization treatment conditions and the washing and signal amplification steps to achieve optimal results (Table 1). Prior to the ISH assay, slides were baked for 30 min at 60 °C. A hydrophobic barrier was created around the sections with an ImmEdge hydrophobic barrier pen (Biozol diagnostica Vertrieb GmbH, Eching, Germany). Sections were treated with hydrogen peroxide for 10 min at RT to inhibit endogenous peroxidases. A second pretreatment step was performed by incubation of the tissue sections in target retrieval buffer maintained at boiling temperature (100 °C to 102 °C) for 5 or 15 min, a step required for breaking crosslinks introduced upon fixation. The tissue sections were completely dried overnight (RT) and treated with a broad-spectrum Protease Plus solution at 40 °C to make the RNA accessible. The CBSV-Mo83 probes were hybridized at 40 °C in a HybEZ II oven for 2 h. As a control, the CBSV-Mo83 probe was hybridized with sections prepared from mock inoculated/grafted plants. Probe hybridization was followed by serial amplification steps (AMP1 to AMP6), as recommended by ACD, testing varying times (10, 15 or 30 min) for the AMP5 step. All washing steps following hybridization and during amplification consisted of two/three incubations in washing buffer (provided with the kit) for 5 min at each step. A final hybridization step using an alkaline phosphatase-labelled probe was followed by incubation with Fast-Red substrate that resulted in red precipitates. Slides were washed in water, counterstained with 50% hematoxylin (Sigma-Aldrich) for 2 min, then rinsed several times in distilled water. Sections were dried at 60 °C for 45 min, submerged in xylene, covered with EcoMount mounting media (Biocare Medical, Pacheco, CA, USA) and with 24x50mm microcover glass. Once mounted, the sections were air-dried for at least 10 min at RT prior to imaging.

**Table 1:** RNAscope® in situ hybridization conditions tested for protocol definition

Sample	Pretreatment conditions			Probe concentration	AMP5 incubation time	Washes after probe and AMPs incubation	Substrate incubation	Observations
	Target Retrieval	Hydrogen Peroxide	Protease Plus					
*N. rustica* (stem)	15 min	10 min	30 min	not diluted	30 min	2 W, 2 min each	10 min	Strong background
	5 min	10 min	10 min	not diluted	15 min	3 W, 5 min each	2 min	Background reduced but low signal
	15 min	10 min	15 min	not diluted	15 min	3 W, 5 min each	2 min	Significant background
	15 min	10 min	15 min	not diluted	10 min	3 W, 5 min each	2 min	Significant background
	15 min	10 min	15 min	1:10 in diluent buffer	10 min	3 W, 5 min each	2 min	Minimal background
	15 min	10 min	15 min	1:40 in diluent buffer	10 min	3 W, 5 min each	2 min	Minimal background
Cassava (stem)	15 min	10 min	15 min	not diluted	10 min	3 W, 5 min each	8 min	Absence of background
	15 min	10 min	15 min	not diluted	15 min	3 W, 5 min each	8 min	Absence of background
Cassava (leaf, petiole)	15 min	10 min	15 min	not diluted	10 min	3 W, 5 min each	8 min	Absence of background
	15 min	10 min	15 min	not diluted	15 min	3 W, 5 min each	8 min	Absence of background

### Imaging

Imaging of the tissue sections was performed using an Olympus SZX16 stereomicroscope (Olympus Deutschland GmbH, Hamburg, Germany) and a Zeiss Axioscope.A1 (Zeiss, Jena, Germany). To improve imaging, the “D” setting of the modulator disk of the Askioscope.A1 was also used for acquisitions in dark field. Fluorescence microscopy was performed using a Leica SP8 confocal microscope (Leica Microsystems, Wetzlar, Germany) with a 20X/0.75 IMM objective using the following settings: 561 nm excitation and 631–651 nm emission window. Images from transmitted light were also collected during the acquisitions to allow overlay of different channels. Images were processed using the Huygens deconvolution software (Scientific Volume Imaging, Hilversum, The Netherlands).

## Results and discussion

Localizing viruses in host tissues and organs can provide fundamental details on virus infection processes as well as on the host responses to virus invasion. Immunohistochemistry (IHC) and ISH are powerful techniques that allow the detection of target molecules in tissues and cells. ISH was originally developed to localize specific DNA sequences on chromosomes and later adapted using various probe modifications and labels to detect mRNAs and other RNA targets [25, 26]. ISH has been extensively used for in situ studies of plant DNA and RNA viruses in different hosts, and simple, rapid and inexpensive methods allow localization of viruses in plants and in insect vectors [27–39].

**Fig. 1:**
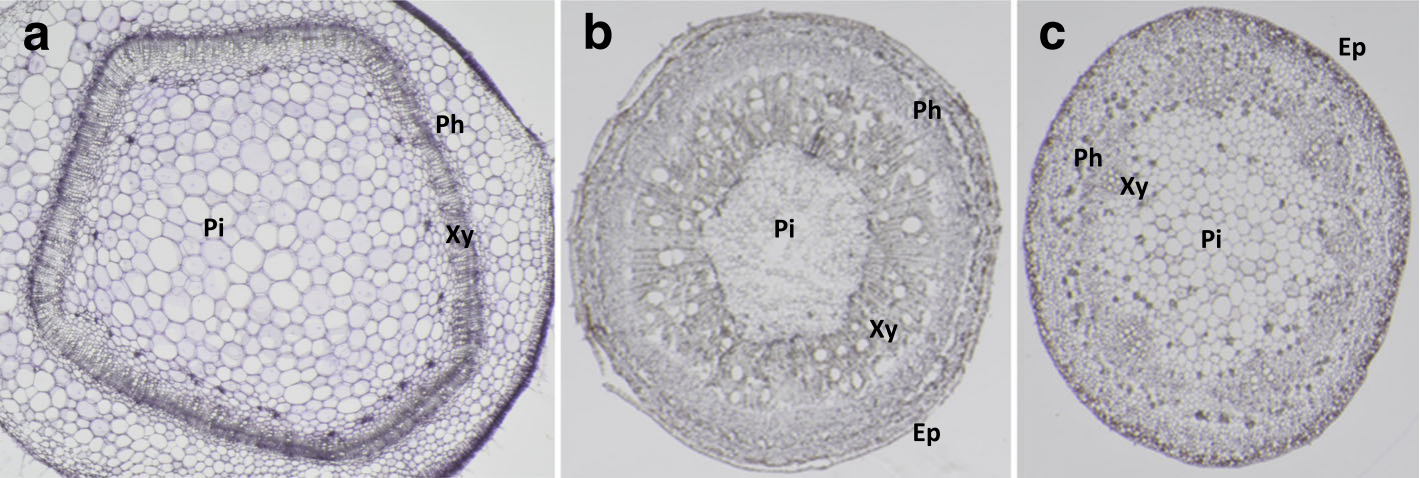
Sections of *Nicotiana rustica* and cassava tissues. Cross-section of *N. rustica* stem tissues (**a**), cross-section of cassava stem tissues (**b**), cross-section of cassava petiole tissues (**c**). Tissue sections were stained by hematoxylin. Ep, Epidermis; Pi, pith; Xy, xylem; Ph, phloem. Sections diameter: stems, ~ 4 mm; petiole, ~ 1.5 mm

Because an in situ hybridization method for CBSV RNA has not been described, we developed an ISH protocol based on RNAscope®. Infection of CBSV-Mo83 in *N. rustica* resulted in stunting and leaf curling and chlorosis, while infected cassava plants showed typical brown streak symptoms on the stems and leaf chlorosis. In the two hosts, semi-thin cross sections of 10–15 μm were obtained using different embedding media. Stem sections of *N. rustica* were prepared from low-melting agarose-embedded tissues (Fig. 1), because this method was straightforward and did not require handling of hazardous chemicals. For cassava tissue samples, the low-melting agarose did not provide sufficient mechanical support to obtain sections of the desired thickness, as stem tissues have hard external cores and a soft internal medulla that present abrupt changes of resistance to the cutting knife, resulting in shattered cuts. The low-melting wax-based sectioning also did not result in satisfactory sections because of shredding and tearing of the wax ribbons, producing only partial, fragmentary sections. Finally, the paraffin-based method was successful for sectioning of cassava tissues, resulting in consistent and uniform sections for all tissue types (Fig. 1) and, therefore, was chosen for the ISH experiments.

**Fig. 2:**
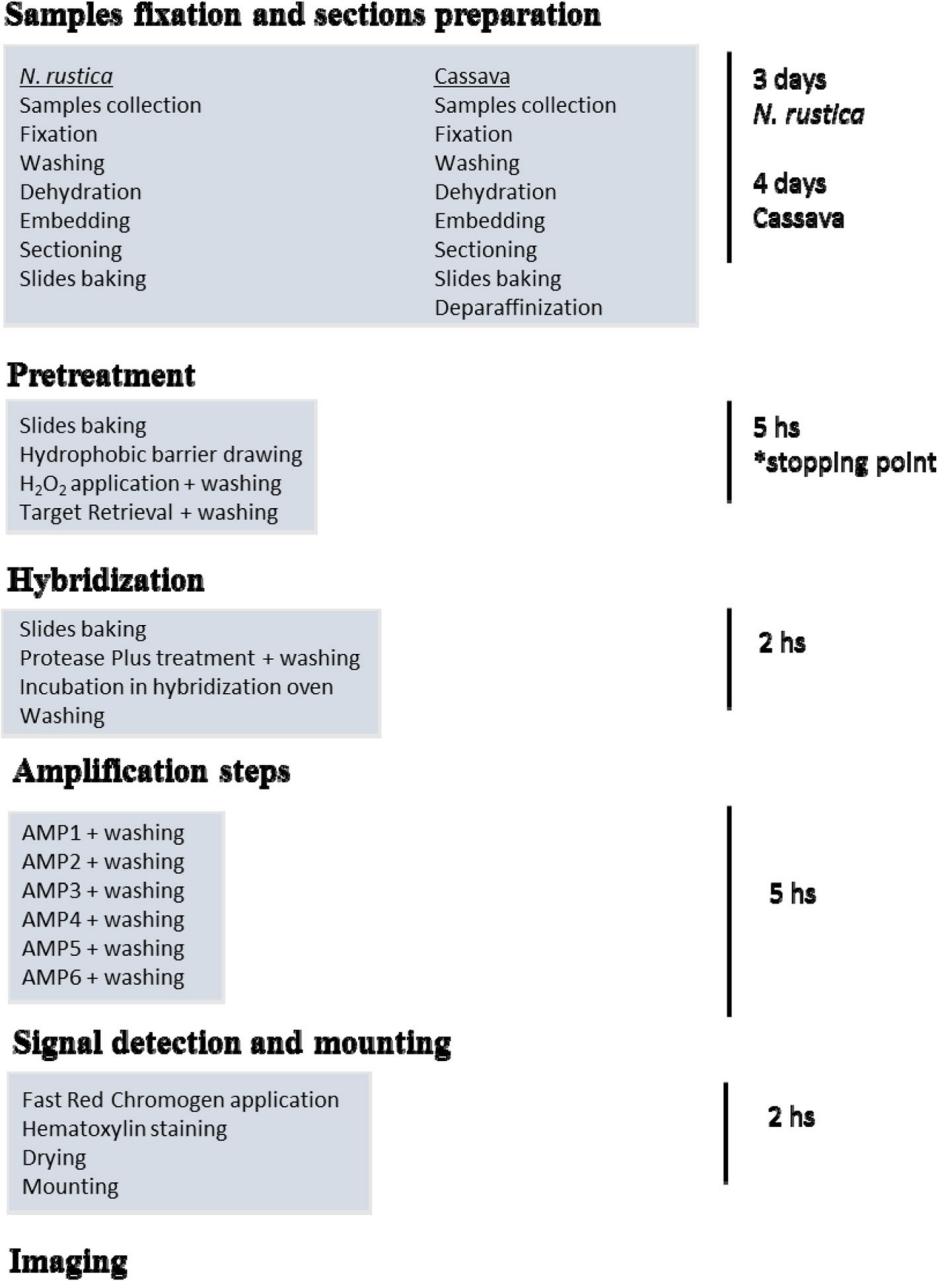
Overview of the RNAscope® protocol for CBSV ISH in *N. rustica* and cassava tissues

The ISH assay conditions were first optimized for CBSV detection in *N. rustica* and subsequently adapted for cassava experimentation. Table 1 summarizes the different ISH assay conditions tested. Key steps in the baseline ACD protocol were modified, including target retrieval, protease treatment, probe concentration, amplification steps, washing and substrate incubation. We found that reducing the protease incubation time and the AMP5 step and increasing the washing steps resulted in a significant reduction of the background. The concentration of the probes was also critical in *N. rustica* section hybridization, wherein 1:10 and 1:40 dilution of the stock provided within the kit resulted in minimal background in infected sections and no signal from healthy controls. In cassava, hybridizations were performed using undiluted probes, showing that the optimal probe concentration needs to be determined for each specific virus/host combination. The optimized incubation conditions for *N. rustica*, also applicable to ISH of cassava tissues, consisted of a 15 min target retrieval pretreatment, 10 min peroxidase treatment, 15 min protease incubation and 10 min AMP5 incubation. The optimal substrate reaction lasted 2 min for *N. rustica* and 8 min for cassava. The final protocol is summarized in Fig. 2 and allowed detection of CBSV RNA in the tissue sections of both hosts, without signal in healthy controls. The conditions determined for ISH are well-suited for sensitive and specific RNA detection, and the protocol provides a good reference for investigating other virus/host combinations.

**Fig. 3:**
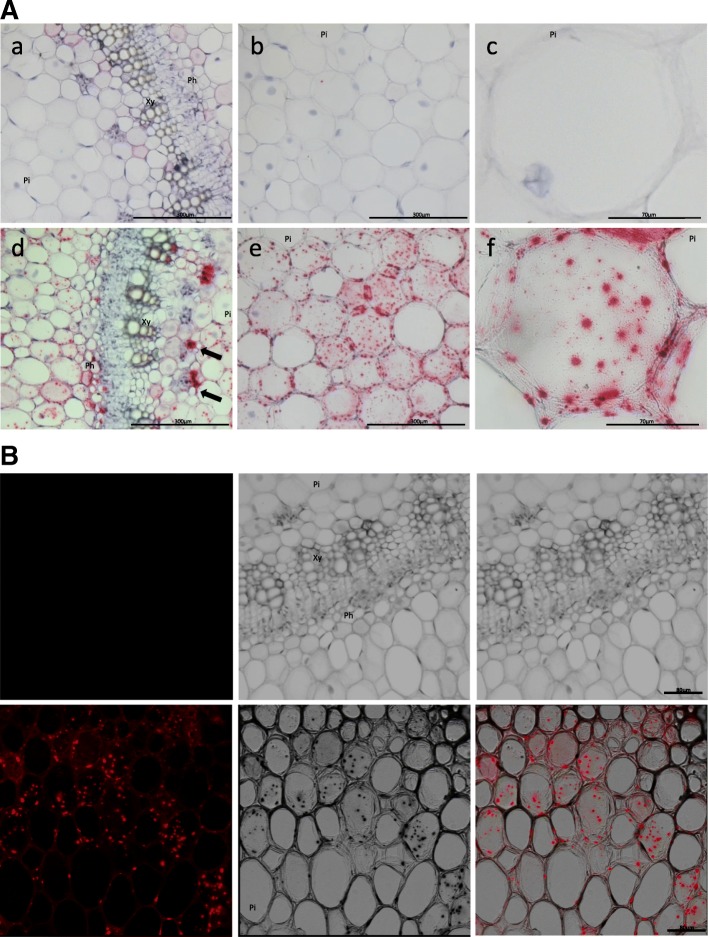
Localization of CBSV-Mo83 RNA in cross-sections of *Nicotiana rustica* stem tissues. **A** Acquisitions using light microscopy. Upper images: tissue from healthy control plants. Lower images: tissue from plants infected with CBSV-Mo83. Abundant presence of viral RNA in cells is visualized as single red dots or dots merged into signal clusters (arrows). **B** Acquisition by confocal laser scanning microscopy. Upper images: non-infected control. Lower images: CBSV-Mo83-infected section. The Fast-Red signal (left) laid over the image from transmitted light (centre) results in a merged picture (right) to demonstrate virus detection in infected cells. Pi, pith; Xy, xylem; Ph, phloem

**Fig. 4:**
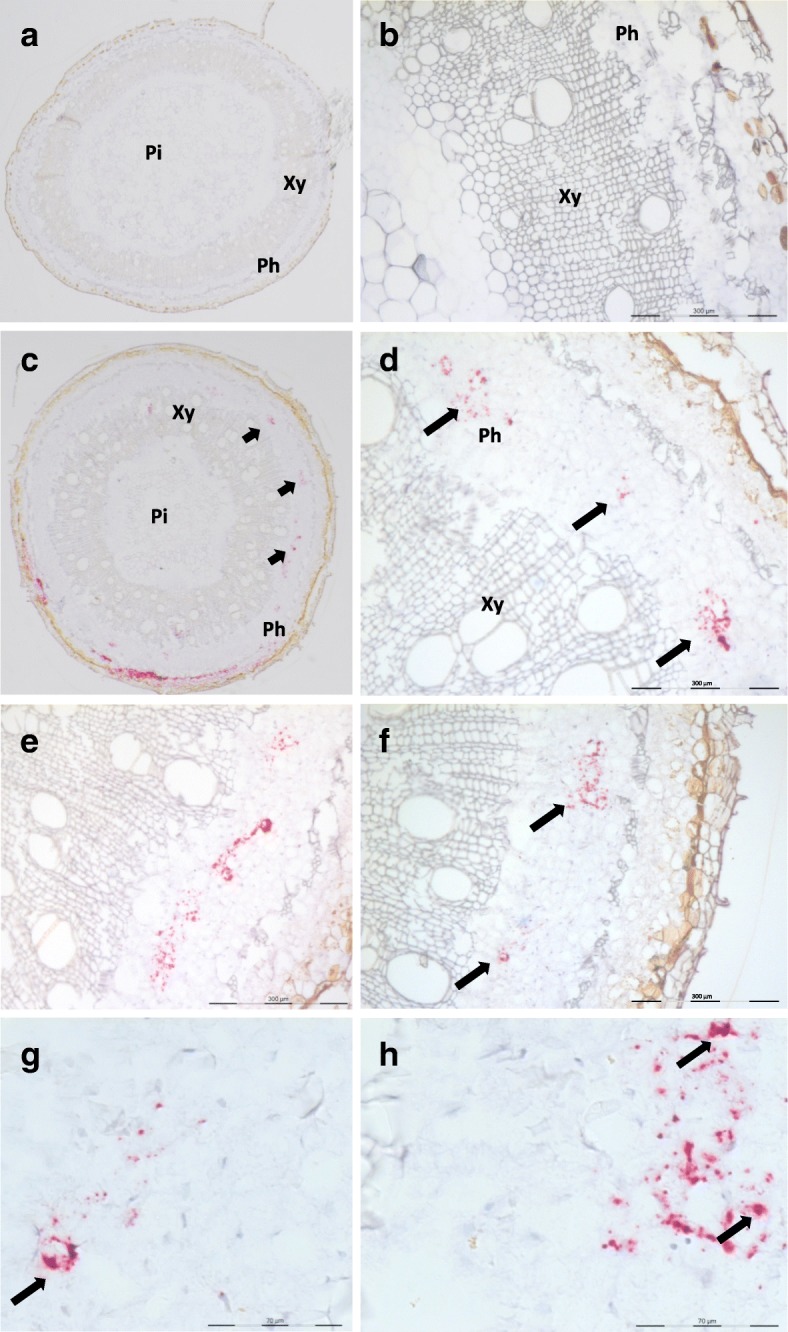
Localization of CBSV-Mo83 RNA in cross-sections of cassava stem tissues. Sections from a control healthy plant (**a**, **b**), stem sections from a CBSV-Mo83-infected cassava (**c**-**h**). Red dots indicate hybridization of the probes to viral RNA. Accumulation of dots mostly in phloematic cells (arrows in **c**, **d** ,**f**). Signal clusters were also detected (**g**, **h**). Pi, pith; Xy, xylem; Ph, phloem. Sections diameter: ~ 4 mm

**Fig. 5:**
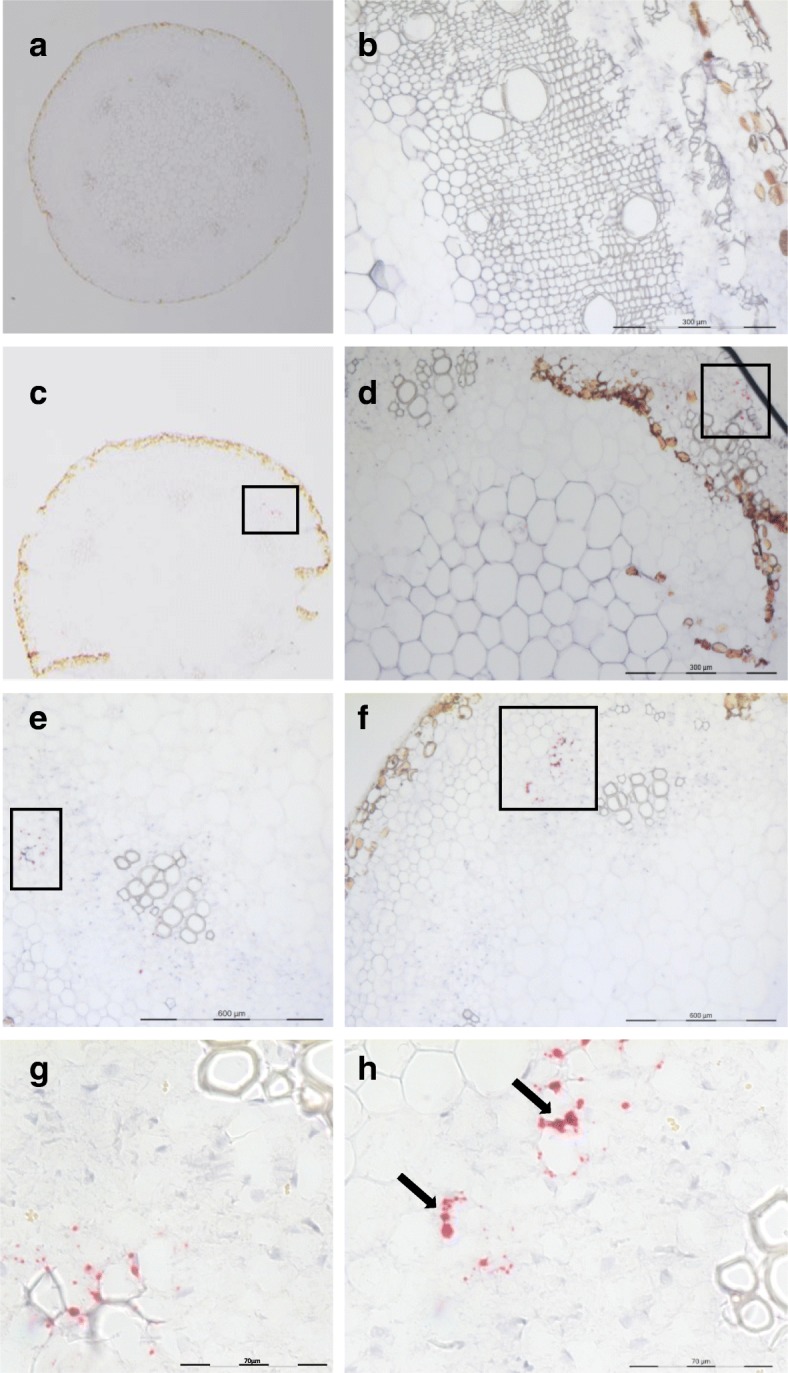
Localization of CBSV-Mo83 RNA in cross-sections of cassava petioles. Petiole sections from healthy control plants (**a**, **b**); sections from CBSV-Mo83-infected cassava (**c**-**h**). Red dots indicate hybridization of probes to viral RNA. Open squares indicate areas with hybridization signals (**c**-**f**), shown in lower panels (**g**, **h**) at a higher magnification. Accumulation of red dots is mostly evident in phloematic tissues. Signal clusters were also detected (**h**). Sections diameter: ~ 1.5 mm

**Fig. 6:**
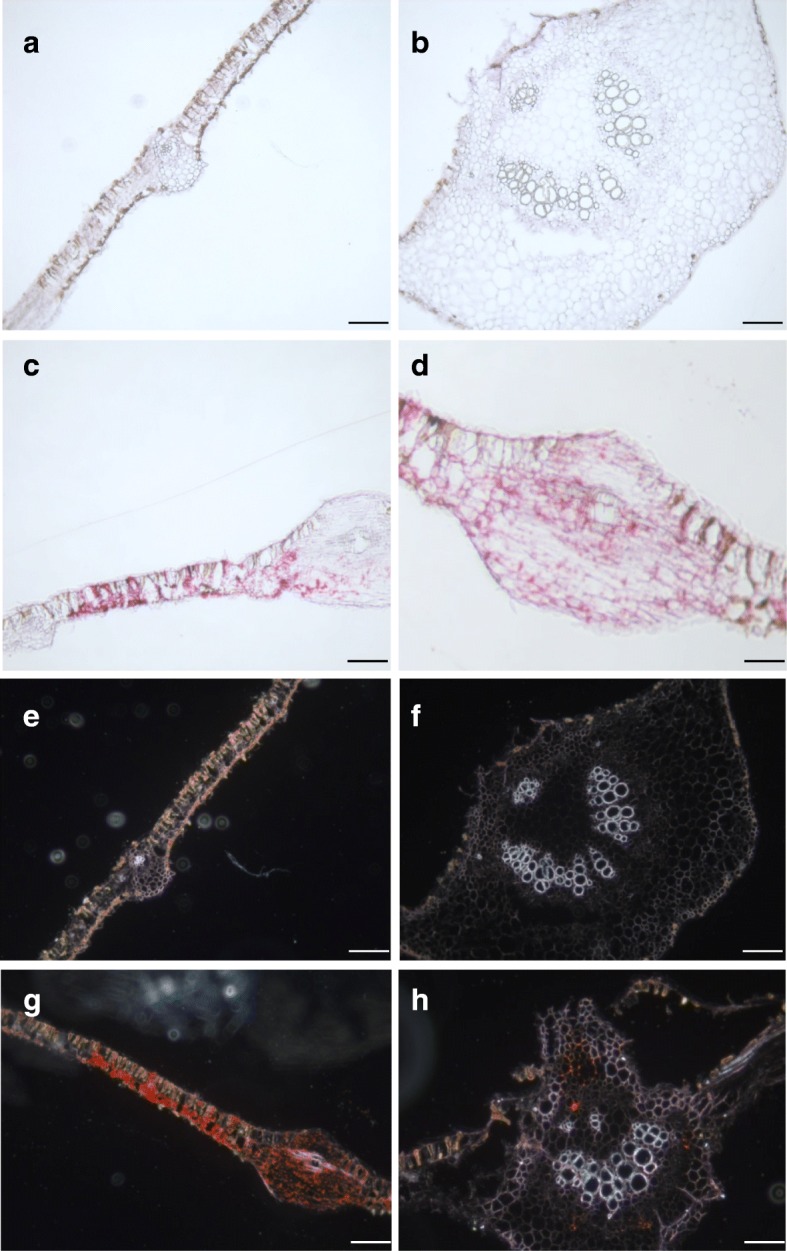
Localization of CBSV-Mo83 RNA in cross-sections of cassava leaves. Lamina and midrib tissues of healthy (**a**, **b**, **e**, **f**) and CBSV-infected leaves (**c**, **d**, **g**, **h**). Hybridization signals (evident as red dots or bright orange dots) show an abundant presence of viral RNA in infected cells, with an absence of signal in non-infected controls. Scale bars: 100 μm

Imaging of at least 10 sections for each condition revealed that CBSV RNA was widely distributed throughout the stem tissues of infected *N. rustica,* as indicated by distinct red dots in different tissues, including the phloem, cortex, and pith cells, which occasionally formed clusters (Fig. 3A, panels d,e,f). The red signal was completely absent in healthy controls (Fig. 3A, panels a,b,c). Since the chromogenic red precipitate can be imaged by fluorescence microscopy, we examined the sections using confocal laser scanning microscopy, and CBSV RNA could be very clearly visualized (Fig. 3B). In cross-sections of infected cassava stem tissues, the red dot signal was less abundant and typically appeared as clusters of dots in phloematic tissue surrounding the xylem (Fig. 4c-h, arrows) and occasionally in cortical tissues (Fig. 4c). There was no signal detected in sections of the healthy controls, indicating absence of any background due to non-specific hybridization (Fig. 4a,b). In cross-sections of infected cassava leaf petioles, CBSV RNA was associated with phloematic tissues (Fig. 5c-f, open squares), and there was no signal detected in the healthy controls. In cross-sections of infected cassava leaves, viral RNA was detected in palisade, mesophyll and midrib tissues (Fig. 6c,d), while there was no signal in leaves of non-infected plants (Fig. 6a,b). To improve signal detection, we examined the sections using dark field acquisition settings, which significantly improved the signal detection and were particularly useful to detect signals in sections with a low number of chromogenic spots (Fig. 6g,h).

Overall, a preliminary examination of CBSV-infected stem sections from *N. rustica* and cassava showed that viral RNA was highly abundant in the phloematic and non-phloematic tissues of *N. rustica*, while in cassava, CBSV RNA appeared more localized around phloematic tissues. It now remains to be investigated whether this difference was because of a considerably lower virus load in cassava compared to *N. rustica* or was a result of a different tissue tropism, as has also been shown for other viruses infecting cassava [39]. While further studies are pending, our results show that the ISH RNAscope® assay has the high resolution required to study virus invasion in cassava cultivars with differential responses to virus infection. The unique probe design and high resolution of RNAscope® also allows detection of multiple targets, as shown previously for two distinct citrus tristeza virus strains in double-infected plants [24]. We are now investigating mixed infections between cassava brown streak virus species and strains as well as mixed infections between CBSV and viruses causing cassava mosaic disease. It will be interesting to further combine RNAscope® ISH with IHC [40–42] to reach a more complete representation of the tissues and the interacting partner molecules.

## Conclusions

We provide a protocol for the detection and localization of CBSV in tissue sections of *N. rustica* and cassava using a highly sensitive ISH technique based on RNAscope® technology. The assay allows in situ hybridization of CBSV RNA in different plant tissues and provides a platform to investigate CBSV tropism during plant infection and disease development.

## Abbreviations

CBSD: Cassava brown streak disease
CBSV: Cassava brown streak virus
DEPC: Diethylpyrocarbonate
DIECA: Diethyldithiocarbamic acid
DSMZ: Deutsche Sammlung von Mikroorganismen und Zellkulturen
EDTA: Ethylenediaminetetraacetic acid
ELISA: Enzyme-linked immunosorbent assay
IHC: Immunohistochemistry
ISH: In situ hybridization
mRNA: Messenger RNA
RT-PCR: Reverse-transcription polymerase chain reaction
UCBSV: Ugandan cassava brown streak virus
